# Diet to Data: Validation of a Bias-Mitigating Nutritional Screener Using Assembly Theory

**DOI:** 10.3390/nu17152459

**Published:** 2025-07-28

**Authors:** O’Connell C. Penrose, Phillip J. Gross, Hardeep Singh, Ania Izabela Rynarzewska, Crystal Ayazo, Louise Jones

**Affiliations:** Northeast Georgia Medical Center, Gainesville, GA 30501, USA; phillip.gross@nghs.com (P.J.G.); hardeep.singh@nghs.com (H.S.); ania.rynarzew@gmail.com (A.I.R.); crystal.ayazo@nghs.com (C.A.);

**Keywords:** dietary complexity, assembly theory, food behavior, GARD screener, ultra-processed foods, fasting, microbiome, dietary assessment, nutrition screening

## Abstract

**Background/Objectives:** Traditional dietary screeners face significant limitations: they rely on subjective self-reporting, average intake estimates, and are influenced by a participant’s awareness of being observed—each of which can distort results. These factors reduce both accuracy and reproducibility. The Guide Against Age-Related Disease (GARD) addresses these issues by applying Assembly Theory to objectively quantify food and food behavior (FFB) complexity. This study aims to validate the GARD as a structured, bias-resistant tool for dietary assessment in clinical and research settings. **Methods:** The GARD survey was administered in an internal medicine clinic within a suburban hospital system in the southeastern U.S. The tool assessed six daily eating windows, scoring high-complexity FFBs (e.g., fresh plants, social eating, fasting) as +1 and low-complexity FFBs (e.g., ultra-processed foods, refined ingredients, distracted eating) as –1. To minimize bias, patients were unaware of scoring criteria and reported only what they ate the previous day, avoiding broad averages. A computer algorithm then scored responses based on complexity, independent of dietary guidelines. Internal (face, convergent, and discriminant) validity was assessed using Spearman rho correlations. **Results:** Face validation showed high inter-rater agreement using predefined Assembly Index (A_i_) and Copy Number (N_i_) thresholds. Positive correlations were found between high-complexity diets and behaviors (rho = 0.533–0.565, *p* < 0.001), while opposing constructs showed moderate negative correlations (rho = –0.363 to −0.425, *p* < 0.05). GARD scores aligned with established diet patterns: Mediterranean diets averaged +22; Standard American Diet averaged −10.

## 1. Introduction

### 1.1. What Is the Guide Against Age Related Disease (GARD)?

Does the body treat 100 calories of potatoes the same way it treats 100 calories of crackers? Conventional dietary assessments might say yes—tallying macronutrients and calories between foods as if they were always metabolically equivalent. But emerging research suggests otherwise: the *structure* of food—its molecular complexity and behavioral context—can shape metabolism, insulin sensitivity, satiety, microbiome health, and long-term disease risk in dramatically different ways [1,2,3].

This paper introduces and validates the *Guide Against Age-Related Disease* (GARD) screener, a new tool that quantifies diet and eating behavior complexity using principles from Assembly Theory. The GARD distinguishes between high-complexity foods (e.g., fresh plants, fermented foods, farm-direct proteins) and low-complexity foods (e.g., ultra-processed items, refined ingredients, distracted eating), scoring them based on quantifiable molecular and behavioral complexity.

High-complexity diets, as determined by the GARD, are consistently associated with better metabolic health, insulin sensitivity, cognitive resilience, microbiome diversity, and lower rates of chronic disease [4,5]. In contrast, diets measured as low-complexity—common in ultra-processed food environments—correlate with overeating, inflammation, insulin resistance, and gut dysbiosis [6,7,8,9]. Yet, traditional screeners focus primarily on gross estimates of intake—particularly fruit and vegetable consumption—without accounting for behavioral complexity or molecular food differences [10,11]. The GARD assesses both what people eat and how they eat—whether meals are shared or solitary, mindful or distracted, outdoors or inside—assigning scores that reflect the real-world complexity of eating. In this study, we detail the design, administration, and validation of the GARD screener, demonstrating its ability to distinguish between healthy and unhealthy eating patterns across molecular, behavioral, and statistical dimensions.

Quantifying diet and eating behavior is essential for advancing research, clinical practice, and public health [12]. The GARD screener is designed to mitigate both recall bias and the Hawthorne effect. Recall bias refers to the systematic error introduced when participants do not accurately remember or report past behaviors—especially common in dietary surveys [13]. To reduce recall bias, the GARD uses a standardized, interviewer-led script to capture a detailed account of the previous day’s intake, which is then scored objectively using principles from Assembly Theory. To address the Hawthorne effect—behavior changes that occur simply because individuals know they are being observed—the GARD blinds participants to the grading criteria and asks only what they ate yesterday. This helps ensure that responses reflect actual behavior, not perceived expectations. The screener was developed to objectively assess not only diet quality but also the behaviors that shape it. Beyond nutrient composition, it captures information about food sources, providing critical insight into dietary health [14]. Likewise, eating behaviors, such as mindful or distracted eating, play a fundamental role and are evaluated alongside food intake [15,16].

Existing frameworks—such as the Penn Healthy Diet Survey—omit both food behaviors while eating and animal protein source [17,18,19]. This omission is a flaw, as behavioral context (e.g., eating socially vs. distracted) and food origin (e.g., farm-direct vs. processed animal product) independently influence metabolic outcomes, satiety signals, and long-term disease risk [3,14]. Without measuring these factors, dietary screeners miss determinants of health beyond macronutrient composition. The GARD may help address this gap by suggesting that the measurable complexity of biological systems offers a meaningful way to assess food and food behaviors (FFB), enabling the GARD to precisely measure nutrition and behavior in terms of complexity.

### 1.2. What Is Assembly Theory and How Does It Apply to Human Health?

Assembly Theory works to measure the complexity of objects using:

- Assembly Index (A_i_)—the smallest number of physical steps needed to construct an object from basic building blocks.
- Copy Number (N_i_)—the number of identical copies of that object in a given environment.

In simple terms, an object is considered *complex* when it takes many steps to build (high A_i_) and is also found in large numbers (high N_i_). For example, molecules with an A_i_ of 15 or more and N_i_ over 10,000 have *only* been observed to originate from biological systems, not from random chemical reactions [20,21,22]. The threshold of 15 steps is grounded in both experimental and theoretical research showing that abiotic (non-living) processes cannot produce such complex molecules by chance [20,23]. A helpful analogy is LEGO^®^ bricks: if every person on Earth were given 15 LEGO pieces at random, the odds of multiple people building the exact same structure are extremely low—unless they are all following a shared plan. In the same way, finding many identical molecules with high A_i_ suggests a directed, living process behind their formation. The 10,000-copy threshold (N_i_) reflects the practical limit of detection in chemical analysis tools like nuclear magnetic resonance (NMR), which require a minimum concentration of identical molecules to register a signal. Therefore, the threshold of 10,000 identical molecules was selected, as this is the minimum number of identical units detectable by nuclear magnetic resonance [24].

Deoxyribonucleic acid (DNA) is an example of a high A_i_ and N_i_ molecule that could only have been created by pre-existent life. Assembly Theory categorizes our genome as consistent of highly complex molecules considering it requires >15 steps to create each individual strand (high A_i_) and exists nearly identically in each of the human body’s 10 billion bone marrow cells (high N_i_) [25,26]. In juxtaposition, atmospheric oxygen (O_2_) would be an example of a simple molecule. While it has a high N_i_ in the atmosphere, it has a low A _i_, often resulting from a <15 step process such as the product of simple inorganic decomposition [27]. For example, the O_2_ on Jupitar’s moon Ganymede is likely from the ultraviolet irradiation of water, not aerobic respiration [28]. Additional examples are shown in Table 1.

Modern Ultra-Processed Food (UPF), as defined by the Nova Classification, is similar to atmospheric oxygen; its components have high N_i_ but low A_i_; they are refined but simple [29]. Refined oils (fats), carbohydrate monomers (sugars), and reconstituted soy bean isolates (proteins) are used to create the plethora of UPF associated with negative health outcomes [14,30]. While these ingredients possess high N_i_ from refining, the complex food matrix is reduced to the constituent building blocks [31]. These foods do not contain the abundance of complex molecules found in whole foods [32]. For instance, while an average supermarket-shelf pastry may contain 40 refined ingredients including artificial preservatives, apples contain hundreds of complex phytochemicals, including a variety of flavonoids, phenolic acids, and antioxidants, which have been linked to various health benefits [33]. This loss of complexity removes plant compounds critical for regulating inflammation, supporting the microbiome, and preventing chronic disease [34,35,36].

### 1.3. How Assembly Theory Can Measure Diet Behaviors

Assembly Theory, while frequently applied to physical objects also extends to abstract constructs that exhibit complexity arising from cumulative interactions and evolving components [20,24]. Fundamentally, Assembly Theory is a study of the extrinsic information needed to discriminate between a given complex object and a random ensemble. This extrinsic information is defined as assembly space; it is the set of all possible construction pathways from irreducible components capturing the minimal steps and the reusable pieces needed to quantify complexity [37]. It follows that as something grows larger, the number of possible ways to arrange its components increases exponentially. For instance, if the assembly space is large enough, it becomes practically impossible to recreate a symphony without an external guiding influence, like the composer. This means that for anything of high complexity (i.e., dance, music, culture, and language), information beyond the observed pattern itself is required for their construction [38,39].

For example, social behavior exhibits greater complexity than passive activities like eating alone while watching television. A sea anemone, which evolved underwater millions of years before humans walked on land, can observe its environment while consuming food but lacks the capacity for nuanced political or emotional discussions—skills derived from components of a large assembly space and that require significantly more assembly time to develop [40]. Assembly time is the total time spent in constructing an object or concept, including the time encoded in the processes and patterns that guide its recreation, rather than just the immediate creation of the object itself [20]. Social engagement is more complex than passive behaviors due to its extensive assembly time and space; it is consistently recreated to develop the intricate interactions and patterns involved in meaningful community connections [41].

The principles of Assembly Theory suggest that behaviors arising from large assembly spaces and long assembly times are fundamentally different from frequent but less intricate behaviors, which rely on smaller assembly spaces and shorter assembly times. For example, social interaction is more complex than passive solitary behaviors because it requires the integration of cognitive, emotional, and social cues in real time, while solitary behaviors involve fewer variables and less cognitive load, as they do not demand the same level of social processing [42]. FFB can also be analyzed through this lens. Certain dietary patterns and eating behaviors require higher levels of structured assembly, distinguishing them from simpler, repetitive consumption habits [43].

Eating behaviors that involve social engagement or environmental variability introduce additional layers of structural and cognitive complexity [43]. By applying Assembly Theory, we can systematically categorize and measure these variations, providing a quantitative framework for understanding the dietary impact on health (Table 2).

In light of all this, we hypothesized that FFB with high complexity, measured by elevated Assembly Index (A_i_) and Copy Index (N_i_), correlated with behaviors and diets known to promote health, enabling the quantification of healthy FFB. This paper outlines the development and validation of the **GARD**, a framework for quantifying FFB complexity using Assembly Theory, establishing a foundation for future studies to assess its relationship between complexity and wellness outcomes.

## 2. Materials and Methods

With the ability to quantify FFB based on Assembly Theory, the GARD was designed to simplify all FFB into two categories: high- and low-complexity (Table 3).

### 2.1. Data Collection Methods

**Figure 1:**
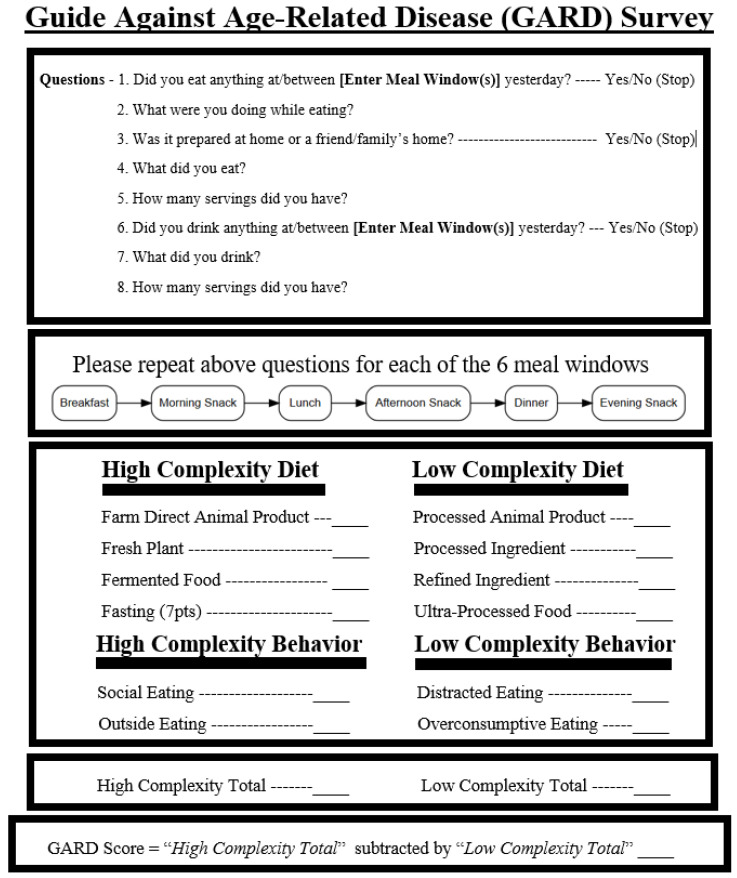
The GARD survey instructions and score card.

A printable survey was designed to quantify the complexity of food consumed and associated food behaviors (see Figure 1). The GARD has two components: collecting a food diary and grading the food diary. Covered below are the different aspects of the survey: collecting the food diary, defining what can be counted as a point, and determining if a point is high- or low-complexity.

### 2.2. Collecting the Food Diary

The survey is most similar to others which quantify abstract concepts like cognition, such as the Montreal Cognitive Assessment (MoCA), in that it requires two people to perform correctly: the provider and the patient [47]. In the GARD, the provider reads through each of the eight questions, stopping at question 8 or stopping if the patient answers “no” to questions 1, 3, or 6. The set of questions is repeated for each of the six daily eating windows to ensure all FFB are accounted for in the diary.

Diets can vary over time; however, most people exhibit stable dietary patterns shaped by culture, convenience, and economic access [48]. As such, a patient who consumes a Standard American Diet (−10 points) on one day is unlikely to switch to a complex Mediterranean-style diet (14–21 points) the next. Empirical reviews support the use of 24 h recalls, showing they provide reliable estimates of diet quality while minimizing recall bias [49]. Because longer recall windows increase error, GARD focuses on the prior day to optimize both accuracy and feasibility [50].

If a patient answers question 3 indicating their food was prepared outside the home, the administrator moves to the next eating window and starts back at question 1. Given not all ingredients (preservatives, food colorants, stabilizers, refined ingredients) are not always readily available to patients when “eating out”, this choice ensures an accurate assessment of food quality by prioritizing meals with fully known ingredients.

Question 4 can require clarification to determine the ingredients in a meal. If a patient states they ate a ham sandwich, the provider must ask what was in the sandwich. Each individually purchased or collected item should receive a point. For example, the sandwich may contain white bread, deli ham, tomatoes, lettuce, and mustard. Each of these five ingredients would receive a point and be categorized as high- or low-complexity. As the patient states all FFB from the prior day, each response is graded according to the criteria below.

### 2.3. Defining a Point

A point is assigned for each individually purchased or collected item. For instance, one picked or purchased tomato is a single item, so it receives a single point. Additionally, one purchased processed pastry is identified as one point despite comprising over 40 ingredients. Whether a point is assigned in the low- or high-complexity category is discussed below.

### 2.4. Grading of High-Complexity Variables

High-complexity markers are characterized by FFB with A_i_ and N_i_. These include fresh plant ingredients, farm-direct animal products, fermented food, and fasting. Additionally, behaviors such as social eating and eating outdoors are considered high-complexity eating behaviors.

#### 2.4.1. Fresh Plant

A point will be given for every unique plant in a patient’s diet. If a patient eats a salad with seven unique plants and spices the provider will tally an additional seven high-complexity points. Note that a type of plant can only be counted once. If a patient eats a tomato for lunch and dinner, that is still 1 point.

A plant can only be counted once because the diversity of plants in the diet—rather than the quantity of any single plant—is the strongest predictor of gut microbiome diversity and complexity. Individuals who consumed at least 30 different types of plants per week have significantly greater microbial complexity compared to those who eat fewer plant types. The microbiome benefits most from a wide range of plant-derived fibers, polyphenols, and prebiotics. Variety, not redundancy, is what drives microbiome diversity and complexity [51].

#### 2.4.2. Farm-Direct Animal Product

A point was given for any eggs, seafoods, or meats the patient ate if the product came from a farmer, rancher, or fisherman known directly by the patient or by someone they know. This was captured by asking patients where they obtained their animal products. If they answered “from a farmer they know,” “their own livestock,” or “wild-caught game,” they received +1 for a healthy animal product. This distinction helps ensure the meat is complex and less affected by long supply chains or added preservatives [52].

Concerning honey, given the presence of enzymes and essential vitamins, natural honey is a part of a high-complexity diet [53].

#### 2.4.3. Fermented Food

An additional point will be given if the plant product is fermented to account for the complexity of the microbiota in the food. For example, sauerkraut has three ingredients: salt, water, and cabbage, which would only be considered one point. However, it is the cabbage’s microbiome that proliferates in the salty brine to give the characteristic tangy taste. This microbiome is another complex ingredient [54]. Therefore, fermented foods receive an additional point to account for the complexity of the microbiota.

#### 2.4.4. Fasting (Autophagy)

Autophagy begins around 12–13 h of fasting, reaching a peak between 18 and 24 h [55]. Since autophagic processes originate from living tissue, they represent a rich source of biological complexity [56]. Patients who intentionally fast for more than 12 h receive 7 points per skipped meal. The assignment of 7 points per skipped meal during fasting was based on the observed average complexity of a high-quality Mediterranean meal (~22 points/day ÷ 3 meals/day ≈ 7 points/meal). This value aligns fasting with the complexity value of a high-complexity meal.

Clinicians should confirm that fasting is a deliberate and appropriate choice, as involuntary food deprivation due to economic or social factors is classified as starvation. For clinical practice, appropriate fasting should be limited to individuals >18 years old and with a body mass index (BMI) > 20 [57]. Fasting is classified as a dietary choice and not a behavior because it changes *what* a person eats, not just the way food is consumed.

#### 2.4.5. Social Eating

A point is given for each meal shared with at least one other person. Social eating introduces unpredictability as conversations, social cues, and external influences shape the experience. Eating in a crowded space without interaction does not qualify for a +1 point. From an evolutionary perspective, the assembly space for social eating is more constrained than solitary eating, as the structures enabling social interaction had to first evolve and be conserved [58].

#### 2.4.6. Outside Eating

A point is given for meals consumed outdoors in natural settings. Exposure to fluctuating environmental conditions—such as temperature, wind, and ambient sounds—adds dynamic complexity, absent in controlled indoor spaces. In evolutionary terms, eating in a dynamic environment represents a larger assembly space than eating in a static one, as homeostatic mechanisms had to evolve to adapt to environmental variability [59].

### 2.5. Grading of Low-Complexity Variables

Low-complexity FFB are characterized by primarily low A_i_. These include processed ingredients, refined ingredients, processed animal products, and ultra-processed foods. Additionally, distracted eating and over-consumptive eating are considered low-complexity.

#### 2.5.1. Processed Ingredients

Processed ingredients are synthesized additives and chemicals absent in whole foods. These include artificial sweeteners (e.g., sucralose), preservatives, emulsifiers, and industrial compounds that extend shelf life, enhance flavor, or modify texture. These additives simplify food complexity. An apple’s flavor and texture arise from hundreds of high A_i_ molecules, while an apple-flavored pastry relies on less than forty low A_i_ molecules [33].

Any store-bought food that contains a processed ingredient is considered low-complexity. However, if a processed ingredient is added to a high-complexity food by personal choice, the food itself remains high-complexity. For example, black coffee earns one high-complexity point. Adding sucralose (Splenda^®^) introduces one low-complexity point, resulting in a net score of zero rather than a negative score.

#### 2.5.2. Refined Ingredients

Refined ingredients are stripped-down versions of whole foods, losing fiber, micronutrients, and bioactive compounds [60]. Examples include white rice, sugar, seed oils, and white flour. These ingredients create uniform, energy-dense products with minimal complexity. Refined sugar in a homemade cookie earns a low-complexity point, as does a store-bought pastry or drink with added sugar.

#### 2.5.3. Processed Animal Products

Processed animal products—such as deli meats, fast-food burgers, and preserved sausages—are shelf-stable, ultra-processed meats sourced from food systems typically three or more steps removed from the consumer. These items undergo extensive modification through curing, salting, or chemical additives [61]. In contrast, farm-direct meat from farmers’ markets, usually pasture-raised and minimally processed, contains significantly higher levels of omega-3 fatty acids, conjugated linoleic acid (CLA), and fat-soluble vitamins A and E due to the animals’ forage-based diets [62,63]. Shorter storage times also help preserve these nutrients by reducing oxidative and enzymatic degradation [64,65]. While processed meat remains chemically dense and highly repetitive at the molecular level, it is less complex overall than farm-direct meat, which exhibits a more diverse and functionally rich molecular structure. Examples of how different meats are graded can be seen below (Table 4).

#### 2.5.4. Ultra-Processed Foods

Defined by the NOVA classification system, ultra-processed foods (UPFs) are made from refined ingredients and industrial additives, using techniques that degrade the natural food matrix. These products lack intact whole foods and are engineered for convenience, shelf stability, and hyper-palatability. Examples include fast food, sugary cereals, packaged snacks, and frozen meals. In the GARD framework, such foods are scored as low-complexity [66].

#### 2.5.5. Distracted Eating

Distracted eating occurs when doing something while eating indoors—driving, working, watching a screen, etc. Lacking social interaction and environmental variability, it happens in controlled, repetitive settings, contrasting with the dynamic nature of social eating [67].

#### 2.5.6. Over-Consumptive Eating

Often associated with UPF, the GARD defines over-consumptive eating as consuming more than one serving in a sitting, prioritizing quantity over diversity. Unlike high-complexity behaviors, which adapt to hunger cues, this behavior disregards them [68]. One cookie earns a low-complexity point for being ultra-processed; multiple cookies earn an additional point for each cookie eaten over the recommended serving size. This overconsumption occurs because UPF’s hyper-palatable formulations stimulate reward pathways and disrupt natural satiety signals [69,70].

### 2.6. Grading the Food Diary

Each item is categorized as high- or low-complexity based on the reasoning found in the research design. A breakdown of how the ham sandwich would be graded can be seen in Table 5.

### 2.7. Survey Distribution

The GARD screener was adapted into an online survey to collect dietary information (a link to the survey is in the supplemental documentation). Participants answered questions for each of the six daily eating and drinking windows with their responses graded. If a meal was prepared at home or by a friend, additional questions identified the individual ingredients, which were then scored. The same process was applied to beverages consumed. The resulting data showed the amount of each variable that was present in a patient’s diet. Use and creation of an online GARD was chosen to shield the study from bias.

After the institutional review board (IRB) exemption of this study, patients in the Internal Medicine Resident Clinic were provided with a link or QR code to complete the GARD as they waited to be seen by a physician. The patients were informed of the study and that their responses would be used for clinical research once patient identity had been de-identified. Patients were allowed to decline participation. Physicians were unaware if the patient had completed the survey and only complete surveys were used for analysis.

### 2.8. Population

The GARD was administered to patients receiving care at an internal medicine residency clinic located within a southeastern U.S. hospital system. The survey was given only to patients greater than 18 years old and who expressed interest in learning more about healthy diet and lifestyle. This clinic primarily serves an underserved population, including individuals who are uninsured or underinsured. The patient population is medically diverse, approximately 27.8% Black or African American, 23.3% Hispanic or Latino, 12.8% Asian, and 36.1% Caucasian [71], with a high burden of chronic illnesses such as diabetes, hypertension, and cardiovascular disease matching national United State averages. Many patients are older adults managing multiple comorbidities. Among those who provided their year of birth, the average age was 53.69 years (SD = 14.5), with participants ranging from 24 to 85 years old. Overall, 71.9% were female and 28.1% were male.

### 2.9. Statistical Analysis

To assess convergent and discriminant validity, we used Spearman rho correlations, following Test Theory principles that suggest that similar constructs should show stronger correlations, while dissimilar constructs should correlate weak or negative correlation [72]. The null hypotheses tested were as follows:

1. There is no significant positive correlation within a patient’s diet between high-complexity diets and high-complexity behaviors (test of convergent validity).
2. There is no significant positive correlation within a patient’s diet between low-complexity diets and low-complexity behaviors (test of convergent validity)
3. There is no significant negative correlation within a patient’s diet between mismatched pairs: high-complexity diets with low-complexity behaviors, and low-complexity diets with high-complexity behaviors (tests of discriminant validity).
4. We expect to reject the nulls for aligned and mismatched pairs.

## 3. Results

The GARD was internally validated using face, convergent, and discriminant validity testing methods. Each individual survey contributed detailed data, totaling 588 individually graded responses—an average of 17.8 per participant—with a final sample size of 33 participants.

### 3.1. Internal Validity

#### 3.1.1. Convergent Validity

An online survey was designed to collect data on patients’ diets and behaviors following the GARD framework. Given the data structure, nonparametric correlations (Spearman rho) were used to test for convergent validity. Analysis of the survey data demonstrated the following:

A strong positive correlation between high-complexity FFB, rho = 0.533, *p* < 0.001.

A strong positive correlation between low-complexity FFB, rho = 0.565, *p* < 0.001.

These results suggest convergent validity, as the Spearman correlations indicate strong positive correlations among constructs of the same complexity and are sufficient to reject the first two null hypotheses.

#### 3.1.2. Discriminant Validity

**Figure 2:**
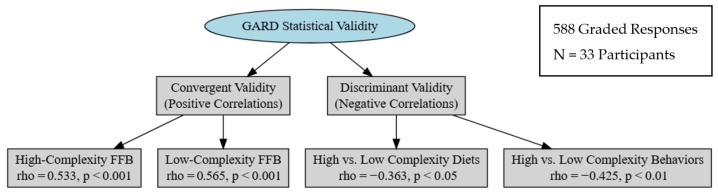
Graphical representation of convergent and discriminant validity.

Similarly to convergent validity testing and given the data structure, nonparametric correlations (Spearman rho) were used to test discriminant validity (Figure 2). Analysis of the survey data demonstrated the following:

A moderate negative correlation between high- and low-complexity diets, rho = −0.363, *p* < 0.05.

A moderate negative correlation between high- and low-complexity behaviors, rho = −0.425, *p* < 0.01.

Given the negative correlations among the discriminant constructs compared to the convergent constructs, discriminant validity is confirmed.

The strong positive correlation between high-complexity diet and high-complexity behavior supports convergent validity. In contrast, the moderate negative correlations between high- and low-complexity constructs—both in diet (rho = –0.363, *p* < 0.05) and behavior (rho = –0.425, *p* < 0.01)—demonstrate discriminant validity, as these relationships are weaker and in the opposite direction compared to the convergent construct. In this light, the final null hypothesis was rejected.

#### 3.1.3. Face Validity

An expert panel was assembled which consisted of four members with diverse and relevant expertise, including a PhD biomedical researcher specializing in diabetes, a PhD statistician, a PhD in health education and survey development, an MD in Family Medicine with a Bachelor of Science in biomedical engineering, and an Exercise Physiologist. Consensus was reached through open discussion, enabling each expert to contribute perspectives until agreement was achieved on key scoring decisions. To establish expert consensus on complexity classification, a grading system was developed to assess the A_i_ and N_i_ of each variable (Table 6, Table 7, Table 8 and Table 9). Since complexity exists on a continuum, the panel agreed on a discrete 1–9 scale to enable consistent, quantifiable scoring. This scale offered sufficient resolution to capture meaningful distinctions. The categories were defined as:

1—Extremely Low

2—Very Low

3—Low

4—Low–Moderate

5—Moderate

6—High–Moderate

7—High

8—Very High

9—Extremely High

The A_i_ and N_i_ scores for each variable were summed with a threshold of 16 points, established for categorizing a high-complexity FFB. This threshold represents the upper third of total scores and aligned with a clear separation in the dataset, where variables rated conceptually as high-complexity consistently scored 16 or above. The cutoff reflects both expert judgment and the natural distribution of the data. Variables scoring 16 or higher received a (+1) GARD score, while those scoring below 16 were assigned a (−1) GARD score.

Given the above scores showing that all high-complexity items meet the cut off of 16 points and the low-complexity items do not meet the cut off, the GARD passed face validation.

### 3.2. Construct Validity

#### Known Group Validity

Construct validity was confirmed by applying the GARD to known healthy and unhealthy diet groups. The GARD consistently assigned high scores to recognized healthy diets and low scores to unhealthy ones, supporting its reliability as a complexity-based dietary assessment tool.

For the GARD to be constructionally valid, it needs to return high scores for established healthy diets and low scores for established unhealthy diets. The chosen healthy diets were the Mediterranean diets and the *National Institute of Health (NIH): Healthy Meal Planning Diet*; both diets have been shown to be beneficial for human health [73,74]. The GARD scores for each of the Daily Meal Plans (three meals without snacks and only drinking water) were able to discriminate between food sources: store vs. farm bought (Table 10 and Table 11).

The GARD was able to evaluate diets established to be unhealthy such as an *80% Ultra-Processed Food Diet* (Table 12) and the *Standard American Diet* (Table 13); all food for these diets was store bought. On average, established unhealthy diets had a lower score than healthy diets.

A detailed breakdown of the grading for each diet, meal, and ingredient across the four dietary patterns is available in Supplementary Materials.

## 4. Discussion

The GARD provides a novel framework for understanding food complexity beyond traditional nutritional assessments. While conventional models focus on macronutrient content and caloric density, the GARD applies Assembly Theory to emphasize molecular structure and organizational complexity [20]. Humans continuously regenerate at the molecular level, yet maintain a consistent identity through a persistent structured pattern [77]. Similarly, food maintains essential patterns of macro- and micronutrients—containing fiber, phytochemicals, and microbiota, all of which contribute to health outcomes [78,79,80]. Assembly Index (A_i_) and Copy Number (N_i_) offer new ways to distinguish between foods that appear similar under conventional classifications. For example, both crackers and potatoes are high in carbohydrates, but a potato’s high A_i_ extensively branched Amylopectin structure is more complex than a cracker’s low A_i_ monomers [81]. This distinction highlights the potential for the GARD to refine dietary assessments and differentiate between nutrient-dense and ultra-processed foods.

The GARD provides a structured method to quantify FFB, which offers potential applications in both research and clinical practice. By distinguishing between high- and low-complexity diets, it could serve as a tool for dietary intervention studies, personalized nutrition counseling, and public health assessments.

In clinical practice, the GARD can track changes in a patient’s diet over time, providing an objective measure of dietary improvement or decline. Many patients perceive their diet as healthy but fail to recognize the actual poor quality of their diet [82]. By assigning a quantifiable score, the GARD offers a concrete number helping patients understand the true impact of their food choices.

While the GARD is still an early-stage tool that requires further validation, it could eventually support state and federal efforts to prioritize access to high-complexity foods. In the long term, this might include guiding how subsidies are allocated, shifting support from low-complexity ultra-processed foods toward local farmers’ markets. Such strategies could help improve population health by making nutritionally rich, complex foods more accessible to underserved communities, though this remains a future possibility contingent on additional evidence.

Future research should explore GARD’s predictive value, assessing whether higher dietary complexity scores correlate with improved health outcomes over time Additionally, integrating the GARD with biomarker analysis, such as Hemoglobin A1C, could further establish its utility as a dietary assessment tool in metabolic and aging research. For instance, future studies could describe if there was a correlation between increased GARD score and reduced Hemoglobin A1C (Table 14).

The GARD uniquely quantifies eating behaviors by recognizing that shared, mindful meals significantly enhance overall health. By measuring factors such as eating together and focusing attention during meals—rather than eating while distracted—the GARD captures essential elements of dietary patterns that traditional assessments often overlook. Social and mindful eating behaviors are particularly impactful. Studies link shared meals with improved nutrient intake [83], while distracted eating has been associated with reduced self-regulation and worse metabolic health. [84]. The GARD incorporates these evidence-based principles by objectively measuring eating behaviors such as social engagement and attention during meals.

Additionally, the GARD mitigates recall bias by having individuals recount only the previous day’s food intake, while healthcare providers objectively assess and assign a dietary grade based on standardized data. This process should reduce subjectivity and the Hawthorne effect, as patients are blinded to the grading criteria.

Moreover, the GARD provides patients with a tangible numerical score that serves as an achievable target for incremental dietary improvement. By focusing on small, consistent changes—such as increasing a score from 2 to 3—patients are encouraged to develop sustainable habits, rather than attempting rapid, large-scale dietary overhauls that frequently fail. This gradual approach harnesses the power of incremental change, leading to lasting improvements in diet and overall health.

Overall, despite a relatively small sample size (*n* = 33), the correlations observed were statistically significant with strong effect sizes (convergent validity, *p* < 0.001). This may be partly attributable to the richness of the dataset, which included 588 individually graded responses—an average of 14.3 per participant. These findings suggest meaningful associations between GARD constructs and support both convergent and discriminant validity. The low *p*-values, even in a limited sample, underscore the robustness of the observed relationships, though further validation in larger and more diverse populations is warranted.

### Limitations

When predicting overall dietary health, GARD assumes that dietary complexity exhibits low variability, implying that most individuals maintain a consistent level of complexity from day to day. This might not be true and may overlook natural fluctuations in eating patterns. Future studies employing month-long food diaries could better assess daily variability in GARD scores.

Although a previous-day recall minimizes recall bias compared to longer recall periods, self-reported dietary data remains inherently prone to errors such as underreporting or overreporting, particularly for unhealthy or socially undesirable foods. Memory limitations, misestimation of portion sizes, and unconscious omissions further compromise data accuracy. To enhance reliability, future studies could examine whether awareness of grading criteria influences recall accuracy, given patients are unaware of how the GARD is graded.

An additional limitation of the GARD framework is its grounding in Assembly Theory, which, though under scientific scrutiny, rests on well-established physics [85]. While more research is needed to link A_i_ and N_i_ directly to health outcomes, this does not undermine our approach. The core premise holds: only biological processes have been observed to reproduce both high A_i_ and N_i_ complexity, supporting our choices of high-complexity variables [20]. This underscores the need for further empirical work to apply these solid theoretical foundations to nutrition. We see this study as an important first step in using quantifiable complexity to advance dietary assessment.

Additionally, while the study demonstrated strong internal and convergent validity, its external validity is limited by the small sample size (*n* = 33) and the single-site setting. Participants were recruited from an internal medicine clinic serving a socioeconomically underserved population in the metropolitan region outside Atlanta, GA. This area is characterized by both economic disparity and substantial ethnic diversity; for example, nearby counties such as Gwinnett and DeKalb report populations that are approximately 27.8% Black or African American, 23.3% Hispanic or Latino, and 12.8% Asian [71]. This intersection of cultural diversity and economic constraint reflects the demographic complexity of many suburban U.S. communities, but may not capture the full range of geographic, socioeconomic, or cultural influences on diet and behavior. Future research in more varied settings is needed to assess the generalizability of GARD scores [86].

## 5. Conclusions

This study introduces the **GARD** as a tool for quantifying dietary and behavioral complexity using Assembly Theory. By applying A_i_ and N_i_ as measures of structured complexity, the GARD successfully distinguishes between high- and low-complexity FFB, aligning with established healthy and unhealthy dietary patterns.

The validation process demonstrates strong internal validity, with predefined complexity classifications aligning with expert consensus, and construct validity, as the GARD reliably scored known healthy diet groups higher than unhealthy groups. The strong correlations observed between high-complexity diets and behaviors, as well as between low-complexity diets and behaviors, further support the framework’s utility in assessing FFB.

While promising, further validation across diverse populations is needed to enhance the GARD’s applicability. Future research should explore how GARD scores correlate with long-term health outcomes and whether interventions based on increasing dietary complexity can predict or improve wellness. By providing a structured method to quantify FFB complexity, the GARD represents a step toward a more systematic approach to understanding the relationship between diet, behavior, and health.

## Figures and Tables

**Table 1:** Different examples of high and low Assembly Index and Copy Number.

**High Assembly Index (A**_i_ **) and High Copy Number (N**_i_ **)**	
DNA polymerase	Enzyme with a complex sequence and local geometry (high A_i_) Replicated billions of times in biology (high N_i_)
Apples	Biological structure: cells, tissues, proteins, pigments (high A _i_) Harvested globally (high N_i_)
The English language	Evolved over thousands of years and formed by thousands of words (high A_i_) Recreated with little variation globally (high N_i_)
**High Assembly Index (A**_i_ **) and Low Copy Number (N**_i_ **)**	
Experimental protein designs	Complex sequence and local geometry (high A_i_) Novel Molecule after first synthesis (low N_i_)
Hand-crafted pastry	Complex structure involving multiple layers, fillings, and precise techniques (high A_i_) Produced in small batches by artisanal bakers (low N_i_)
The word “alacrity”	Thousands of years of culture to create the word (high A_i_) Infrequently used (Low A_i_)
**Low Assembly Index (A**_i_ **) and High Copy Number (N**_i_ **)**	
Water (H_2_O)	Often a biproduct on single step organic reactions (Low A_i_) Found universally in high abundance (high N_i_)
High fructose corn syrup	Fructose molecules refined from a source which initially required <15 steps to assemble the carbohydrate (Low A_i_); * Produced globally for sweeteners (high N_i_)
The sound of a rock falling on impact	Created by a single step process (low Ai) Occurs universally (high Ni)
**Low Assembly Index (A**_i_ **) and Low Copy Number (N**_i_ **)**	
Nitric oxide radical (NO•)	Simple molecule often a biproduct (Low A_i_) Reactive and short-lived (Low N_i_)
Snowball	Formed by aggregation of ice crystals via simple mechanical action (Low A_i_) Individually formed and short-lived (Low N_i_)

* Refining does not increase A_i_, as A_i_ represents the minimal number of steps needed to build an object using reusable parts.

**Table 2:** Examples of behavioral complexity.

Examples of Behavioral Complexity			
Social Eating *	Value	Distracted Eating	Value
Eating a meal with family and/or friends	(+1)	Any eating while watching television	(−1)
Eating a meal while at an indoor soccer game	(+1)	Eating during a lecture	(−1)
Eating a meal while periodically petting your dog	(+1)	Eating while diving	(−1)
Outside Eating	Value	Over-Consumptive Eating	Value
Eating while on a park bench alone	(+1)	Eating more servings than on the nutrition label	(−1)
Eating while at an outdoor soccer game	(+1)	Eating to the point of discomfort	(−1)
Eating strawberries while harvesting	(+1)	Drinking more than 4 standard drinks in an evening	(−1)

* These three activities characterize eating behaviors in the pre and early Neolithic period: (1) eating with others in our tribe [44]; (2) eating while sharing a common goal, such as hunting [45]; (3) eating with a hunting dog [46].

**Table 3:** Quantifiable food and food behavior categories.

Quantifiable Food and Food Behavior Categories			
High-Complexity Diet	Point Value	Low-Complexity Diet	Point Value
Farm-Direct Animal Product	(+1)	Processed Animal Product	(−1)
Fresh Plants	(+1)	Processed Ingredient	(−1)
Fermented Foods	(+1)	Refined Ingredient	(−1)
Fasting (Autophagy)	(+7)	Ultra-Processed Food	(−1)
High-Complexity Behavior	Point Value	Low-Complexity Behavior	Point Value
Social Eating	(+1)	Distracted Eating	(−1)
Outside Eating	(+1)	Over-consumptive Eating	(−1)

**Table 4:** Complexity based off of degrees of separation.

Complexity Based Off of Degrees of Separation			
2 or less degrees of separation	Value	3 or more degrees of separation	Value
Deer meat the patient hunted	(+1)	Packaged meat from a store	(−1)
Chicken eggs from a friend	(+1)	Deli meat from the supermarket	(−1)
Meat from the farmer’s market	(+1)	Barbeque from a restaurant	(−1)

**Table 5:** Example of how the GARD would score a generic ham sandwich.

Complexity Grade for a Generic Ham Sandwich			
Ingredient	Variable	Complexity Grade	Point Value
White Bread	Refined Ingredient	Low	(−1)
Deli Ham	Processed Animal Product	Low	(−1)
Mustard	Processed Ingredient (Food Dye)	Low	(−1)
Tomato	Fresh Plant	High	(+1)
Lettuce	Fresh Plant	High	(+1)

GARD Score = “High-Complexity Total” subtracted by “Low-Complexity Total” = (−1).

**Table 6:** Expert consensus on complexity scores for food categories.

Category	Variable	A_i_ (Assembly Index) of Average Molecule	N_i_ (Copy Number) of Average Molecule	Total	GARD Score
High Complexity	**Fresh Plant**	9 (Extremely High)	9 (Extremely High)	18	(+1)
	**Farm-Direct** **Product**	9 (Extremely High)	9 (Extremely High)	18	(+1)
	**Fermented Food**	9 (Extremely High)	9 (Extremely High)	18	(+1)
	**Autophagy (Fasting)**	9 (Extremely High)	9 (Extremely High)	18	(+1)
Low Complexity	**Processed Ingredient**	3 (Low)	8 (Very High)	11	(−1)
	**Refined Ingredient**	2 (Very Low)	9 (Extremely High)	11	(−1)
	**Processed Animal Product**	7 (High)	8 (Very High)	15	(−1)
	**Ultra-Processed Food**	1 (Extremely Low)	9 (Extremely High)	10	(−1)

**Table 7:** Explanation of expert consensus on complexity scores for food categories.

Category	Variable	Internal Validity in Assembly Theory
High Complexity	**Fresh Plant**	Fresh plants contain diverse, complex biomolecules (e.g., polyphenols, fibers) requiring many synthetic steps (extremely high A_i_), with widespread repetition in plant tissue (extremely high N_i_).
	**Farm-Direct Animal Product**	Whole animal products (meat, seafood) have structured proteins, fats, carbohydrates, and nucleic acids (extremely high A_i_) with considerable repetition within a given tissue (extremely high N_i_).
	**Fermented Food**	Fermentation increases biochemical complexity via the presence of microbiotic life (extremely high A_i_) with high molecular repetition within individual bacteria (extremely high N_i_).
	**Autophagy (Fasting)**	Autophagy recycles highly structured biomolecules, organelles, glycogen, fatty acid chains (extremely high A_i_), which exist in repeating cell types with in a tissue (extremely high N_i_).
Low Complexity	**Processed Ingredient**	Processed ingredients are designed for easy manufacturing (low A_i_) at large volume (very high N_i_)
	**Refined Ingredient**	Industrial processing simplifies molecular structure taking complex biomolecules (i.e., Amylopectin) and turning them into simplified molecules (i.e., Glucose, Fructose) (low A_i_) while increasing uniformity and repetition (very high N_i_).
	**Processed Animal Product**	**Research shows measurable differences in nutritional complexity between processed meat and farm-direct, pasture-raised meat.** Specifically, meat from farmers’ markets—typically pasture-raised and minimally handled—contains significantly higher levels of omega-3 fatty acids, conjugated linoleic acid (CLA), and fat-soluble vitamins like A and E [62,63]. These nutrients result from the animals’ forage-based diets and shorter storage times, which help preserve delicate compounds and vitamins [64,65]. In contrast, processed meat, while chemically dense and uniform, lacks this diversity and freshness. As a result, processed meat exhibits **contain less complex molecules (high A_i_)** compared to farm-direct meat, which contains a more varied and functionally rich molecular structure. However, processed meat still retains a high number of repeated molecules throughout the tissues (Very High N_i_).
	**Ultra-Processed Food**	Ultra-processed foods by definition contain manufactured ingredients (extremely low A_i_) and refined ingredients that are mass-produced and highly repetitive (extremely high N_i_).

**Table 8:** Expert consensus on complexity scores for food behaviors.

Category	Variable	A_i_ (Assembly Index) of Average Molecule	Ni (Copy Number) of Average Molecule	Total	GARD Score
High-Complexity Behavior	**Social Eating**	8 (High)	8 (High)	16	(+1)
	**Outside Eating**	8 (High)	8 (High)	16	(+1)
Low-Complexity Behavior	**Distracted Eating**	4 (Low)	9 (Extremely High)	13	(−1)
	**Over-Indulgent Eating**	3 (Very Low)	9 (Extremely High)	12	(−1)

**Table 9:** Explanation of expert consensus on complexity scores for food behaviors.

Category	Internal Validity in Assembly Theory
High-Complexity Behavior	Social eating requires complex social structures, communication, and shared rituals (high A_i_). It has been a fundamental aspect of human evolution across cultures and time (high N_i_).
	Eating in dynamic outdoor environments requires physiological adaptation to variable conditions (high Ai). This behavior was the norm for most of human history (high N_i_).
Low-Complexity Behavior	Eating while distracted lacks engagement with environmental and social cues, reducing behavioral complexity (low Ai). It is a modern behavior that has become widespread (extremely high N_i_).
	Overeating prioritizes quantity over adaptive responses to hunger and social context, diminishing behavioral complexity (very low A_i_). Engineered food environments have made it exceedingly common (extremely high N_i_).

**Table 10:** GARD scores for daily meal plans based on the *NIH: Healthy Meal Planning* [73].

NIH: Healthy Meal Planning: Tips for Older Adults		
	GARD Score	
Daily Diet (Total GARD score for 3 Meals)	Store Bought	Farm Bought All Homemade
Daily Meal Plan 1	16	27
Daily Meal Plan 2	14	22
Daily Meal Plan 3	12	25
Average	14	23

**Table 11:** GARD Scores for daily meal plans based on the Mediterranean diet [74].

GARD Score for Daily Mediterranean Diets		
	GARD Score	
Daily Diet (Total GARD score for 3 Meals)	Store Bought	Farm Bought All Homemade
Daily Meal Plan 1	19	27
Daily Meal Plan 2	17	22
Daily Meal Plan 3	7	13
Average	14	21

**Table 12:** GARD scores for daily meal plans based on an 80% UPF diet [75].

80% Ultra-Processed Food Diet		
	GARD Score	
Daily Diet (Total GARD score for 3 Meals)	Store Bought	Farm Bought All Homemade
Daily Meal Plan 1	1	N/A
Daily Meal Plan 2	−10	
Daily Meal Plan 3	1	
Total	−1	

**Table 13:** GARD scores for daily meal plans based on the *Standard American Diet* [76].

Standard American Diet		
	GARD Score	
Daily Diet (Total GARD score for 3 Meals)	Store Bought	Farm Bought All Homemade
Daily Meal Plan 1	−12	N/A
Daily Meal Plan 2	−8	
Daily Meal Plan 3	−9	
Average	−10	

**Table 14:** Proposed GARD score correlations for future study.

Proposed GARD Score Correlations for Future Study	
Proposed High GARD Score Biomarker Correlations	Proposed Low GARD Score Biomarker Correlations
Lower fasting insulin levels	Lower levels of Vitamin D
Lower average blood sugar (Hemoglobin A1c)	Lower levels of Cobalamin
Lower Apolipoprotein B (marker of serum lipid levels)	Higher C-reactive protein (CRP)
Proposed High GARD Score Patient Outcome Correlations	Proposed Low GARD Score Patient Outcome Correlations
Lower rates of 30-day hospital readmission	Higher rates of Colon Cancer
Lower quantity of diagnosed chronic conditions	Higher rates of End Stage Renal Disease
Lower patient Body Mass Index (BMI)	Higher rates of Fragility Fractures

## Data Availability

The raw data supporting the conclusions of this article will be made available by the authors on request.

## Supplementary Materials

The following supporting information can be downloaded at https://www.mdpi.com/article/10.3390/nu17152459/s1.

## Abbreviations

The following abbreviations are used in this manuscript:

A_i_	Assembly Index
N_i_	Copy Number
FFB	Food and Food Behavior
GARD	Guide Against Age-Related Disease
UPF	Ultra-Processed Food
MoCA	Montreal Cognitive Assessment
NIH	National Institutes of Health
PUFA	Polyunsaturated Fatty Acid
